# Sirtuin2 suppresses the polarization of regulatory T cells toward T helper 17 cells through repressing the expression of signal transducer and activator of transcription 3 in a mouse colitis model

**DOI:** 10.1002/iid3.1160

**Published:** 2024-02-02

**Authors:** Liuqing Ge, Min Xu, Meifang Huang, Shaoping Liu, Zhidai Zhou, Ziqin Xia, Qiu Zhao, Feng Zhou

**Affiliations:** ^1^ Department of Gastroenterology, Hubei Clinical Center and Key Laboratory for Intestinal and Colorectal Diseases Zhongnan Hospital of Wuhan University Wuhan China; ^2^ Department of Hematology and Oncology, Wuhan Children's Hospital, Tongji Medical College Huazhong University of Science and Technology Wuhan China; ^3^ Medical Research Center Zhongnan Hospital of Wuhan University Wuhan China

**Keywords:** inflammatory bowel disease, regulatory T cells, signal transducer and activator of transcription 3, Sirtuin2, T helper 17 cells

## Abstract

**Introduction:**

Regulatory T cells (Tregs) play an important role in inflammatory bowel diseases (IBDs) through modulating intestinal inflammation. However, the factors affecting Treg function and plasticity during IBD progression are not thoroughly disclosed. The current study aims to reveal new molecular mechanisms affecting Treg plasticity.

**Methods:**

A mouse strain, in which tdTomato and enhanced green fluorescent protein were under the control of the *Foxp3* promoter and *Il17a* promoter, was established and subjected to colitis induction with dextran sulfate sodium. The existence of Tregs and IL‐17‐expressing Tregs (i.e., Treg/T helper 17 [Th17] cells) were observed and sorted from the spleen, mesenteric lymph nodes, and lamina propria by flow cytometry, followed by measuring Sirtuin2 (Sirt2) expression using quantitative reverse transcription polymerase chain reaction and Immunoblotting. Lentivirus‐induced Sirt2 silencing was applied to determine the impact of Sirt2 on Treg polarization to Treg/Th17 cells and even Th17 cells. The effect of Sirt2 on Stat3 was analyzed by flow cytometry and immunoblotting.

**Results:**

Sirt2 was highly expressed in lamina propria Tregs and it moderately suppressed Foxp3 expression as well as the immunosuppressive function of Tregs. Surprisingly, lentivirus‐mediated Sirt2 silencing promoted the generation of Treg/Th17 cells out of Tregs. Sirt2 silencing also enhanced the generation of Th17 cells out of Tregs under the Th17 induction condition. Furthermore, Sirt2 inhibited Th17 induction by suppressing the protein level of the signal transducer and activator of transcription 3.

**Conclusion:**

Sirt2 suppresses Treg function but also inhibits Treg polarization toward Treg/Th17 cells and Th17 cells. The ultimate effect of Sirt2 on colitis might depend on the balance among Tregs, Treg/Th17 cells, and Th17 cells.

## Graphical abstract

Sirtuin2 (Sirt2) is highly expressed in lamina propria Tregs. Sirt2 moderately suppresses Treg function but also inhibits Treg polarization toward Treg/Th17 cells and Th17 cells.

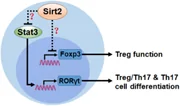

## 1 INTRODUCTION

Inflammatory bowel diseases (IBDs) are characterized as continuous inflammatory responses in the gastrointestinal tract.^1^, ^2^ Both innate and adaptive immune reactions actively participate in the onset and aggravation of IBDs. Multiple immune cell populations, such as macrophages, dendritic cells, T cells, and B cells, play inductive or regulatory roles in the initiation and progression of IBDs.^3^, ^4^, ^5^, ^6^ T cells, especially CD4^+^ T cells, are regarded as the main drivers of IBDs when the intestinal defensive and homeostatic response is disturbed.^5^ Among multiple CD4^+^ T subsets, effector regulatory T cells (Tregs) and T helper 17 (Th17) cells are persistent interest in IBD research, owing to their key role in modulating intestinal inflammation. Featuring the expression of the high‐affinity interleukin (IL)‐2 receptor α‐chain (CD25) and Forkhead box P‐3 (Foxp3), Tregs are indispensable for immune tolerance and homeostasis. Through secreting inhibitory cytokines, interfering with the metabolic processes of inflammatory cells, and neutralizing dendritic cell function, Tregs maintain immune homeostasis at the intestinal mucosal interface.^7^ Th17 cells, however, are considered to disrupt the intestinal mucosa by producing pro‐inflammatory cytokines including IL‐17, IL‐21, and IL‐22.^8^ Importantly, the plasticity of Tregs and Th17 cells allows them to turn to one another. For example, Tregs can polarize into Treg/Th17 cells that co‐express Foxp3, IL‐17, and retinoic‐acid‐receptor‐related orphan nuclear receptor gamma T (RORγt) under the instruction of IL‐6 plus IL‐1β or transforming growth factor‐β (TGF‐β).^9^, ^10^ Nonetheless, the molecular mechanisms responsible for the dynamic plasticity of Tregs in colitis have not been thoroughly elucidated.

Sirtuins are NAD^+^‐dependent deacetylases and/or ADP ribosylases implicated in the modulation of cellular metabolic processes and genetic stability.^11^ Sirtuins also function on the immune system. Sirtuin1 (Sirt1) deficiency promotes T cell activation, compromises T cell immune tolerance, and predisposes an individual to Th17‐related autoimmune disorders.^12^ On the contrary, Sirt1 activation suppresses T cell function.^13^ Moreover, targeting Sirt1 increases Foxp3 expression to augment the immunosuppressive effect of Tregs.^14^ It has been reported that Sirt1 suppresses Foxp3 expression by enhancing Foxp3 degradation and inhibiting the transcriptional activity of Foxp3.^15^, ^16^ Recently, Sirtuin2 (Sirt2) was shown to diminish Foxp3 expression to weaken the anti‐inflammatory effect of Tregs in a cerebral ischemia model.^17^ However, the mechanism by which Sirt2 suppresses Treg function remains unidentified. Moreover, whether Sirt2 influences the Treg/Th17 balance is completely unknown.

In the current study, through characterizing the expression and function of Sirt2 in Tregs in a mouse colitis model, we discovered that Sirt2 was highly expressed in lamina propria Tregs and it suppressed Foxp3 expression while preventing Treg polarization toward Th17 cells, probably through downregulating signal transducer and activator of transcription 3 protein rather than reducing Sirt2 messenger RNA (mRNA). Our study demonstrated the role of Sirt2 in regulating Treg plasticity for the first time.

## 2 MATERIALS AND METHODS

### 2.1 Mice

Upon approval from the Wuhan University Animal Care and Use Committee, the study was implemented under the Wuhan University Animal Use Guidelines (Approval# 11400700245566). Eight‐week‐old male homozygous Foxp3‐IRES‐tdTomato‐2A‐CreERT2 mice (Strain Name C57BL/6‐Foxp3^em4(IRES‐tdT^°^mato‐2A‐CreERT2)Smoc^), in which an IRES‐tdTomato‐2A‐CreERT2 expression cassette was knocked into the Foxp3 gene stop codon site via CRISPR/Cas mediated recombination, were purchased from the Shanghai Model Organisms Center, Inc. Eight‐week‐old female homozygous B‐IL17‐enhanced green fluorescent protein (EGFP) mice (Strain name C57BL/6‐Il17at^tm1^/Bcgen), in which an IRES‐EGFP‐SV40‐polyA signal sequence cassette was inserted after the stop codon of the Il17a alleles, were obtained from the Beijing Biocytogen Co. Ltd. Both strains were on the C57BL/6 background. They were crossed to generate the new Foxp3‐tdTomato‐IL17‐EGFP strain in which Foxp3‐expressing cells became tdTomato‐positive and IL‐17‐expressing cells were EGFP‐positive. The mice were housed in a pathogen‐free condition.

### 2.2 Colitis model

The colitis model was established based on our previous protocol.^18^ Briefly, male Foxp3‐tdTomato‐IL17‐EGFP mice were given orally administered 3% dextran sulfate sodium (DSS; Sigma‐Aldrich) in drinking water for 7 days. The control mice were given drinking water.

### 2.3 Collecting immune cells from spleens, mesenteric lymph nodes, and lamina propria

Mice were then euthanized by CO_2_ inhalation. Spleens were harvested and ground on 70‐µm cell strainers on ice to prepare single‐cell suspensions. The mesenteric lymph nodes were taken and processed the same way. Immune cells in lamina propria were enriched following our previous protocol with modifications.^18^ Briefly, mouse colons were cut into small pieces and digested in the digestion buffer (Roswell Park Memorial Institute [RPMI] 1640 medium containing 4 mM ethylenediaminetetraacetic acid, 2.5 mg/mL Collagenase VIII, 100 U/mL DNase I, 5 mM 4‐(2‐hydroxyethyl)‐1‐piperazineethanesulfonic acid, 1 mM dithiothreitol, and 20% fetal calf serum) for 30 min at 37°C. The digested tissues with the supernatants were passed through a 70‐µm cell strainer to prepare single‐cell suspensions. The single‐cell suspensions were centrifuged at 200*g* for 5 min, and red blood cells were lysed by incubating the cell pellets in the ammonium–chloride–potassium lysis buffer for 3 min at room temperature. All reagents were purchased from Sigma‐Aldrich.

### 2.4 Flow cytometry analysis

The following reagents were ordered from Biolegend: APC/Cy7 anti‐CD3 (17A2), Pacific blue anti‐CD25 (3C7), PE‐Cy7 anti‐CD4 (GK1.5), APC anti‐Ki67 antibody (16A8), and the APC Annexin V Apoptosis Detection Kit with 7‐AAD. Alexa Fluor® 647 antiphospho‐Stat3 antibody (Tyr705, 4/P‐STAT3) and APC anti‐Stat3 (Thr37/46, #7547) were purchased from BD Biosciences. The staining procedures were the same as described in our previous study.^18^ Samples were analyzed on an LSRII flow cytometer or sorted on a FACSAria Flow Cytometer (BD Biosciences).

### 2.5 Reverse transcription and quantitative **polymerase chain reaction** (q‐RT‐PCR)

The reagents were purchased from Thermo Fisher Scientific. RNAs were purified with the Arcturus PicoPure RNA Isolation Kit following the manufacturer's manual. Complementary DNAs (cDNAs) were generated with the SuperScript™ Double‐Stranded cDNA Synthesis Kit. cDNAs were mixed with the SYBR™ Green PCR Master Mix before being loaded on a CFX96 Touch™ Real‐Time PCR Detection System (Bio‐Rad). Please refer to Supporting Information S1: Table 1 for the primer sequences. The mRNA levels of the genes of interest were normalized to the mRNA levels of β‐actin and quantified through the $2^{‐∆∆C_{t}}$ formula.^19^

### 2.6 In vitro culture and treatment

The reagents were purchased from BioLegend. For Treg induction, splenic total CD4^+^ T cells were sorted from the Foxp3‐tdTomato‐IL17‐EGFP by flow cytometry. A 96‐well culture plate was coated with 5 µg/mL anti‐CD3ε antibody (145−2C11) overnight at 4°C. Sorted CD4^+^ T cells were placed into the plate at a density of 1 × 10^6^ cells/mL in the presence of 2 µg/mL soluble anti‐CD28 antibody (37.51), 10 ng/mL IL‐2 (575,402), and 2 ng/mL TGF‐β1 (781,802). Three days later, cells were loaded on the flow cytometer for testing the expression of tdTomato and EGFP.

For Th17 induction, 1 × 10^6^ cells/mL sorted splenic CD4^+^ T cells were seed into the precoated culture plate in the presence of 2 µg/mL soluble anti‐CD28 mAb (BioLegend, 37.51), 20 ng/mL IL‐6 (575,704), 1 ng/mL TGF‐β1 (781,802), 2 ng/mL IL‐23 (589,002), 10 µg/mL anti‐mouse IL‐4 antibody (504,122), and 10 µg/mL anti‐mouse IFN‐γ antibody (505,834). On Day 3, 5 mL of fresh media containing the same concentrations of cytokines and antibodies as used on Day 0. On Day 4, cells were loaded on the flow cytometer for testing the expression of tdTomato and EGFP.

For the lentiviral transduction of lamina propria Tregs, the mouse SIRT2 short hairpin RNA lentiviral particles and control lentivirus were purchased from Santa Cruz Biotechnology. CD4^+^ tdTomato^+^EGFP^−^Tregs were sorted from lamina propria of healthy mice and resuspended in RPMI 1640 medium containing glutaMAX and 10% fetal calf serum at the density of 1 × 10^5^ cells/mL in a 96‐well culture plate (Corning), in the presence of 10 ng/mL rmIL‐2 (R&D Systems). The lentiviral particles were then added into the Treg culture at the multiplicity of infection of 10 overnight in the presence of 5 µg/mL polybrene (Sigma‐Aldrich). The medium was replaced with fresh medium and Tregs were incubated for two additional days in the presence of 10 ng/mL rmIL‐2. The transduced cells were then subjected to two different analyses:

(1) Direct flow cytometry assay detecting the expression of IL‐17 and Foxp3 and other functional properties.
(2) Sorting tdTomato^+^ Tregs by flow cytometry from the transduced cells to exclude non‐Treg cells. After sorting, tdTomato^+^ Tregs were subjected to Th17 induction for 3 days as described above. In some experiments, 10 µM C188‐9 (Sigma‐Aldrich, 573128), which is a potent and selective Stat3 inhibitor, was added into tdTomato^+^ Tregs culture at the start of Th17 induction.

### 2.7 Treg function analysis

Splenic CD4^+^CD25^−^ naïve conventional T cells were sorted from healthy mice by flow cytometry. 1 × 10^6^/mL conventional T cells were labeled with 5 µM CellTrace Violet (Thermo Fisher Scientific) in phosphate‐buffered saline for 20 min in a 37°C water bath, followed by the addition of four volumes of RPMI 1640 medium and centrifugation at 250*g* for 5 min. The conventional T cells were then resuspended in RPMI 1640 medium supplemented with 10% fetal calf serum. After that, 5 × 10^4^ lentivirus‐transduced Tregs and 5 × 10^4^ labeled conventional T cells were mixed and seeded in a 96*‐*well microplate which was precoated with 5 µg/mL anti‐CD3ε antibody. Cells were then incubated for 5 days in the presence of 2 µg/mL soluble anti‐CD28 antibody and 10 ng/mL rmIL‐2. The dilution of CellTrace Violet was assessed by flow cytometry.

### 2.8 Immunoblotting

Cellular proteins were extracted by lysing cells for 30 min in ice‐cold RIPA buffer containing protease inhibitors (Thermo Fisher Scientific). The anti‐Sirt2 antibody (EPR20411‐105, 1:1000), anti‐Stat3 antibody (EPR787Y, 1:2000), and anti‐β‐Actin antibody (Abcam 8226, 1:2000) were purchased from Abcam.

### 2.9 Statistics

Data were shown as mean ± standard deviation. Each experiment was repeated independently three times. The unpaired two‐tailed Student's *t* test and one‐way analysis of variance with Tukey's multiple comparison tests were used to test the statistical significance of data differences, respectively. A *p* < .05 is regarded as statistically significant.

## 3 RESULTS

### 3.1 Validation of the new mouse strain

**Figure 1:**
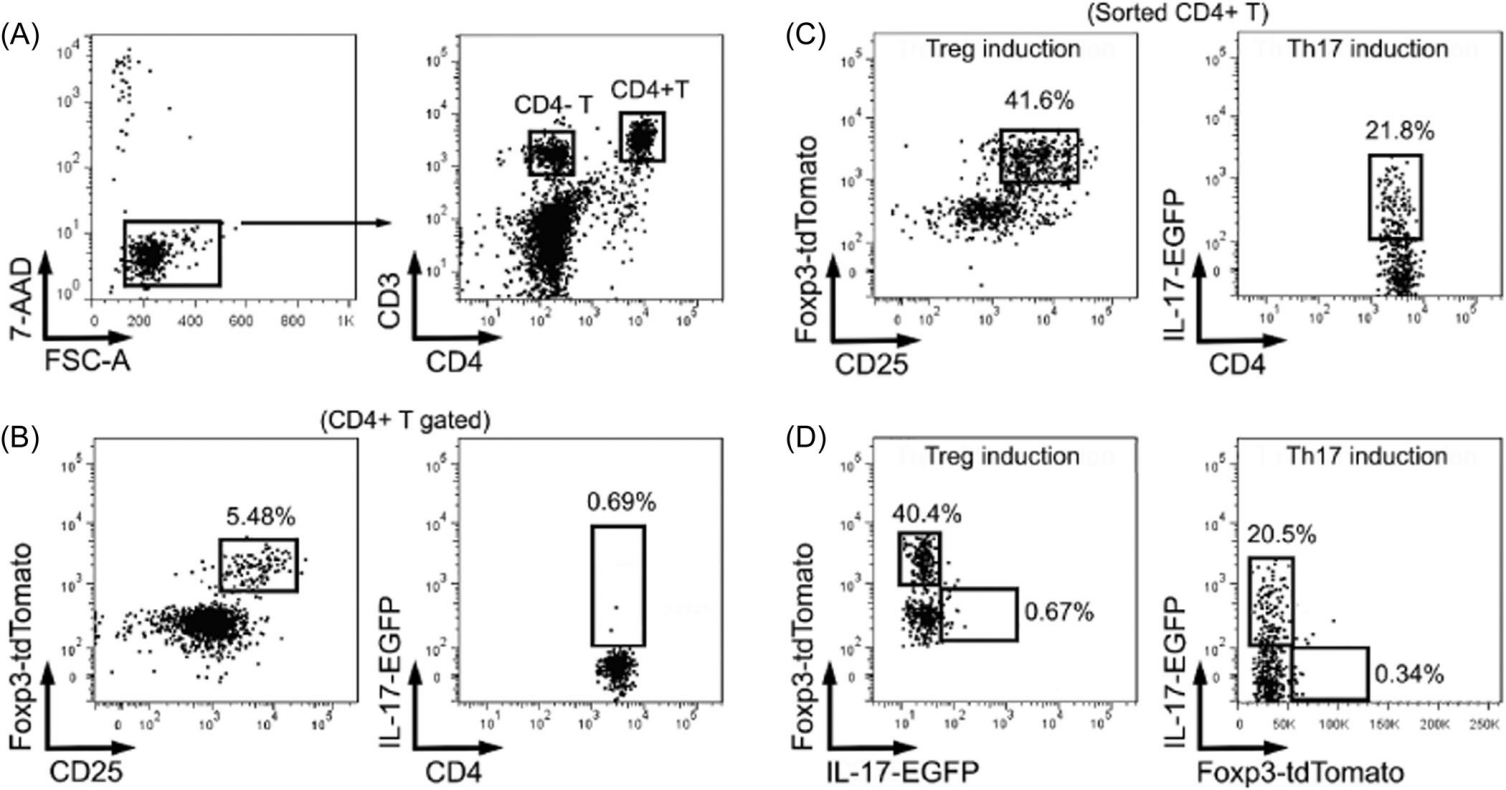
The expression of Foxp3‐tdTomato and interleukin (IL)‐17‐EGFP in regulatory T cells (Tregs) and T helper 17 (Th17) cells of the Foxp3‐tdTomato‐IL17a‐EGFP mouse strain. (A) Identification of 7‐AAD^−^ live cells (left panel) and CD4^+^ T cells (right panel) in splenocytes by flow cytometry. (B) Identification of CD25^+^Foxp3‐tdTomato^+^ Tregs (left panel) and IL‐17‐EGFP^+^ Th17 cells (right panel) in splenic CD4^+^ T cells. (C) Identification of Foxp3‐tdTomato^+^ Tregs (left panel) and IL‐17‐EGFP^+^ Th17 cells (right panel) in sorted splenic CD4^+^ T cells after 3‐day Treg induction using agonistic antibodies and transforming growth factor (TGF)‐β1 (left panel) or 4‐day Th17 induction using agonistic antibodies plus IL‐6, TGF‐β1, IL‐23, neutralizing anti‐IL‐4 antibody, and neutralizing anti‐interferon‐γ antibody (right panel). (D) Co‐expression of Foxp3‐tdTomato and IL‐17‐EGFP in CD4^+^ T cells after Treg induction (left panel) or Th17 induction (right panel). The data represent two independent experiments.

We constructed a new mouse strain by crossing the Foxp3‐IRES‐tdTomato‐2A‐CreERT2 mice with the B‐Il17a‐EGFP mice. The former strain co‐expresses Foxp3 and tdTmato along with CreERT2, while the latter co‐expresses IL‐17 and EGFP. Therefore, the progeny of the two strains, termed Foxp3‐tdTomato‐IL17−EGFP mouse, is expected to produce tdTomato^+^ Tregs and EGFP^+^ Th17 cells. To validate this new strain, we isolated splenic CD4^+^ T cells from the Foxp3‐tdTomato‐IL17−EGFP mice (Figure 1A) to identify CD25^+^tdTomato^+^ Tregs and IL‐17−EGFP^+^ Th17 cells. As shown in Figure 1B, about 5%–6% of splenic CD4^+^ T cells were tdTomato^+^ Tregs while scarce Th17 cells were found. To further determine Tregs and Th17 cells, splenic CD4^+^ T cells were sorted and activated under the Treg induction or Th17 induction condition, respectively. As illustrated in Figure 1C, CD25^+^tdTomato^+^ Tregs accounted for over 40% of total CD4^+^ T cells under the Treg induction condition, whereas over 20% of CD4^+^ T cells became IL‐17−EGFP^+^ Th17 cells under the Th17 induction condition. Moreover, tdTomato^+^ Tregs did not express IL‐17‐EGFP while IL‐17‐EGFP^+^ Th17 cells were almost all tdTomato^−^ (Figure 1D). Hence, Tregs and Th17 cells can be specifically recognized in the new mouse strain.

### 3.2 Sirt2 expression in colitic Tregs

**Figure 2:**
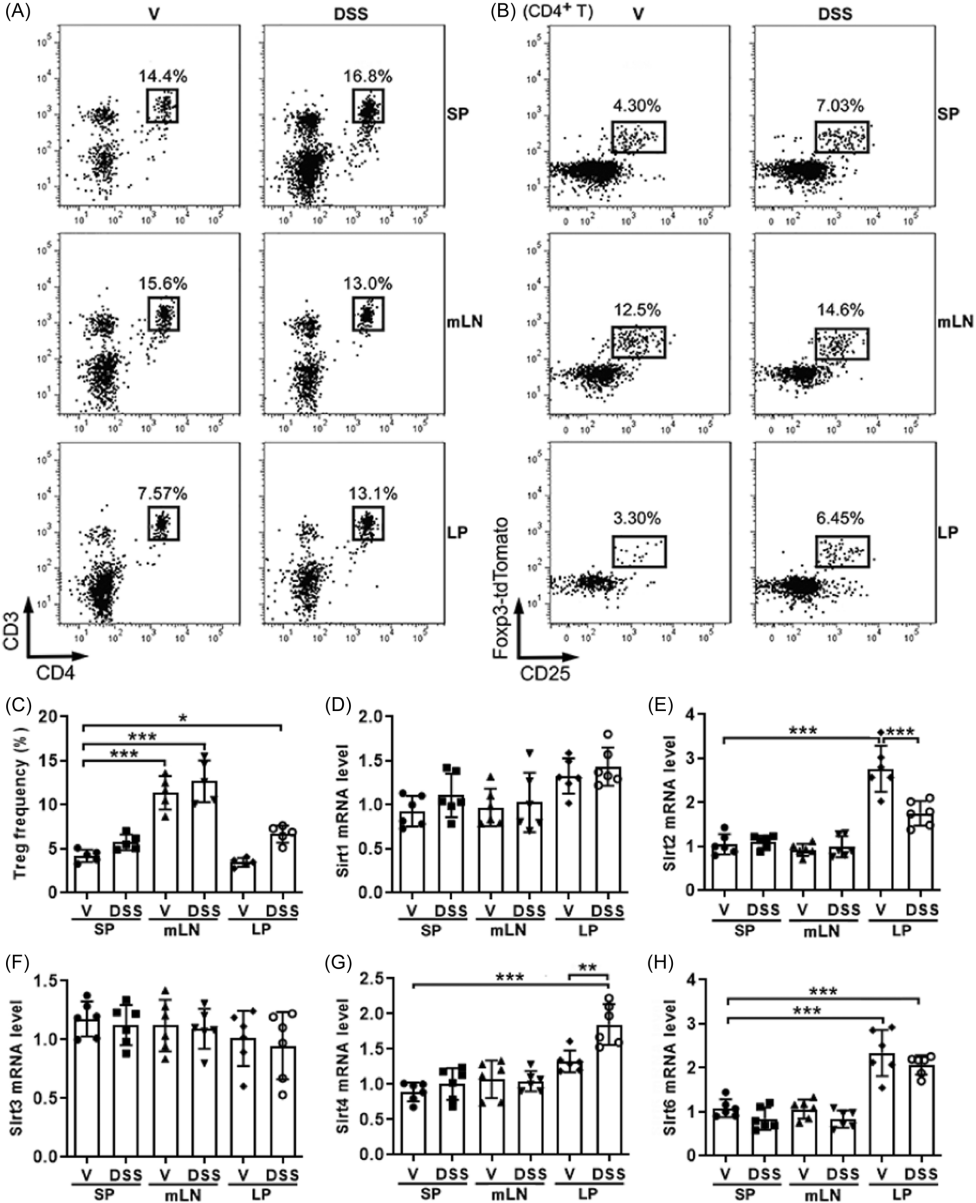
The expression of Sirtuins in regulatory T cells (Tregs). (A) Identification of CD4^+^ T cells in the spleens (SP), mesenteric lymph nodes (mLN), and lamina propria (LP). (B) Identification of Foxp3‐tdTomato^+^ Tregs in CD4^+^ T cells in the SP, mLN, and LP. (C) Frequencies of Foxp3‐tdTomato^+^ Tregs in CD4^+^ T cells in indicated tissues. (D–H) messenger RNA levels of indicated Sirtuins in sorted CD4^+^ tdTomato^+^ Tregs. *N* = 6 per group. *p < .05. **p < .01. ***p < .001. One‐way analysis of variance. DSS, dextran sulfate sodium‐treated colitic mice; V, vehicle‐treated mice.

To determine the significance of Sirtuins to Treg biology in colitis, we induced colitis in Foxp3‐tdTomato‐IL17‐EGFP mice with DSS. After that, CD3^+^CD4^+^ T cells were distinguished in spleens, mesenteric lymph nodes, and lamina propria (Figure 2A). CD4^+^ T cell frequencies were similar in the spleen and mesenteric lymph nodes of healthy and colitic mice. However, CD4^+^ T cell frequency was lower in normal lamina propria but significantly increased in the lamina propria of colitic mice (Supporting Information S1: Figure 1). tdTomato^+^ Tregs were then discriminated and sorted from CD3^+^CD4^+^ T cells (Figure 2B). Although colitis induction did not remarkably alter the frequencies of tdTomato^+^ Tregs in spleens and mesenteric lymph nodes, Treg frequency was increased in lamina propria after colitis induction (Figure 2C). We then sorted CD4^+^ tdTomato^+^ Tregs to measure the mRNA levels of Sirtuins that have been implicated in immunity. The purity of sorted Tregs reached 97%–99% (Supporting Information S1: Figure 2). As demonstrated in Figure 2D,F, the mRNA levels of Sirt1 and Sirt3 were not significantly changed in each tissue after colitis induction. Sirt2, however, was highly expressed in normal lamina propria but downregulated in colitic lamina propria (Figure 2E). Sirt4 expression was upregulated in colitic lamina propria (Figure 2G). Sirt6 was substantially expressed in both normal and colitic lamina propria (Figure 2H).

### 3.3 Sirt2 expression in lamina propria Tregs and Treg/Th17 cells

**Figure 3:**
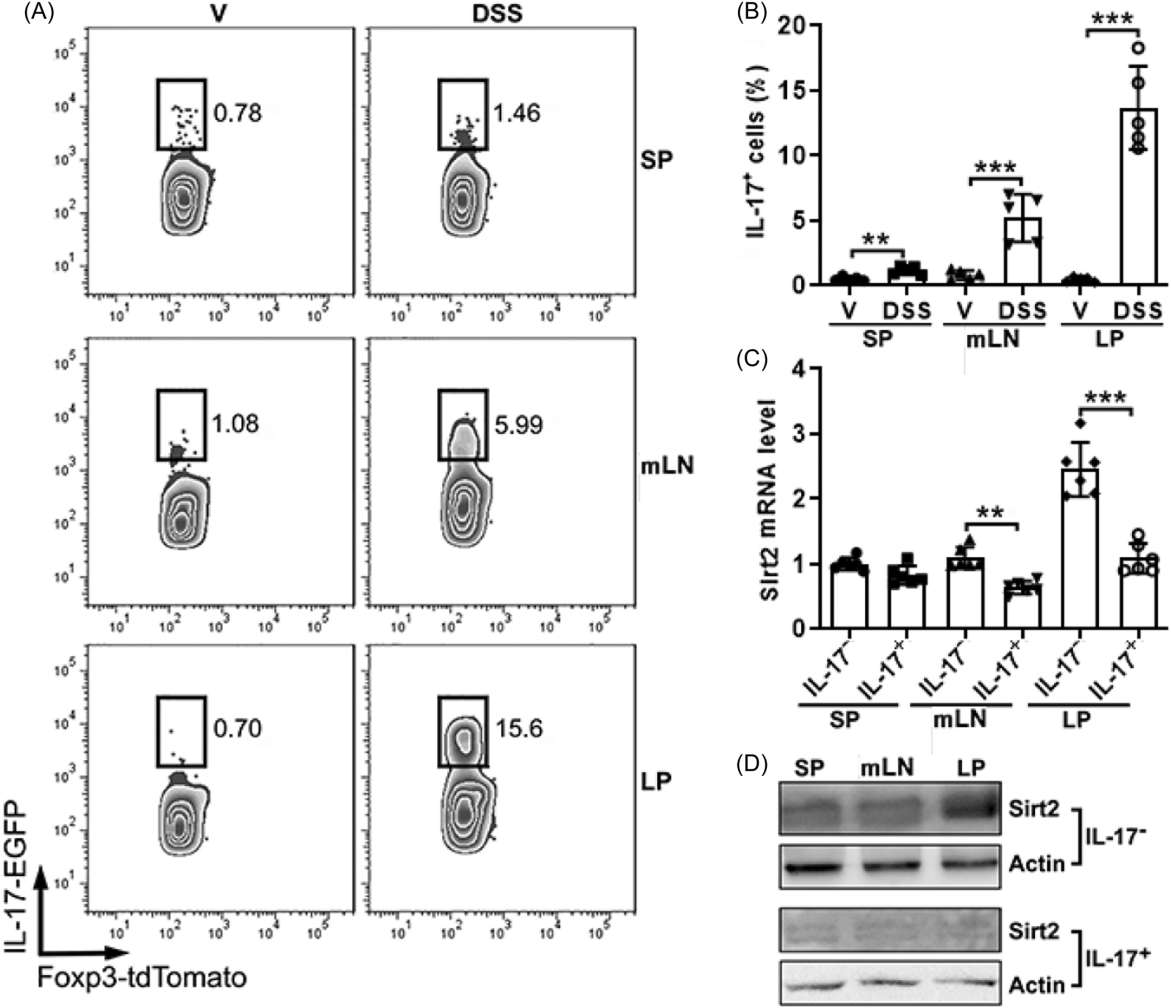
Sirt2 expression in regulatory T cells (Tregs) and Treg/T helper 17 (Th17) cells. (A) Representative flow cytometry zebra plots showing Foxp3‐tdTomato^+^interleukin (IL)‐17‐EGFP^−^ Tregs and Foxp3‐tdTomato^+^IL‐17‐EGFP^+^ Treg/Th17 in the spleens (SP), mesenteric lymph nodes (mLN), and lamina propria (LP). (B) The frequencies of IL‐17‐EGFP^+^ Treg/Th17 cells in indicated tissues. (C) Sirt2 messenger RNA levels in IL‐17‐EGFP^−^ Tregs and IL‐17−EGFP^+^ Treg/Th17 cells that were sorted from colitic mice. (D) Sirt2 protein levels in IL‐17‐EGFP^−^ Tregs and IL‐17‐EGFP^+^ Treg/Th17 cells that were sorted from colitic mice. *N* = five to six per group. **p < .01; ***p < .001. Student's *t* test. DSS, dextran sulfate sodium‐treated colitic mice; IL‐17^−^, IL‐17‐EGFP^−^ Tregs; IL‐17^+^, IL‐17‐EGFP^+^ Treg/Th17 cells; V, vehicle‐treated mice.

We then evaluated the presence of Treg/Th17 cells that co‐expressed Foxp3‐tdTomato and IL‐17‐EGFP in the tdTomato^+^ Tregs. As shown in Figure 3A,B, less than 1% of Treg/Th17 cells were found in the spleens, mesenteric lymph nodes, and lamina propria of vehicle‐treated control mice. In colitic mice, however, the average Treg/Th17 frequency increased to 1.5%, 5%, and 14% in spleens, mesenteric lymph nodes, and lamina propria, respectively. IL‐17^−^Tregs and IL‐17^+^ Treg/Th17 cells were then sorted from colitic mice to quantify Sirt2 expression. In mesenteric lymph nodes and lamina propria of colitic mice, IL‐17^−^Tregs expressed lower Sirt2 mRNA than IL‐17^+^ Treg/Th17 cells (Figure 3C). Immunoblotting showed weak Sirt2 expression in splenic and mLN IL‐17^−^Tregs. However, abundant Sirt2 was expressed in lamina propria IL‐17^−^Tregs (Figure 3D). Meanwhile, Sirt2 protein was also low in IL‐17^+^ Treg/Th17 cells in the spleen, mLNs, and LP (Figure 3D).

### 3.4 The effect of Sirt2 silencing on Treg identity maintenance

**Figure 4:**
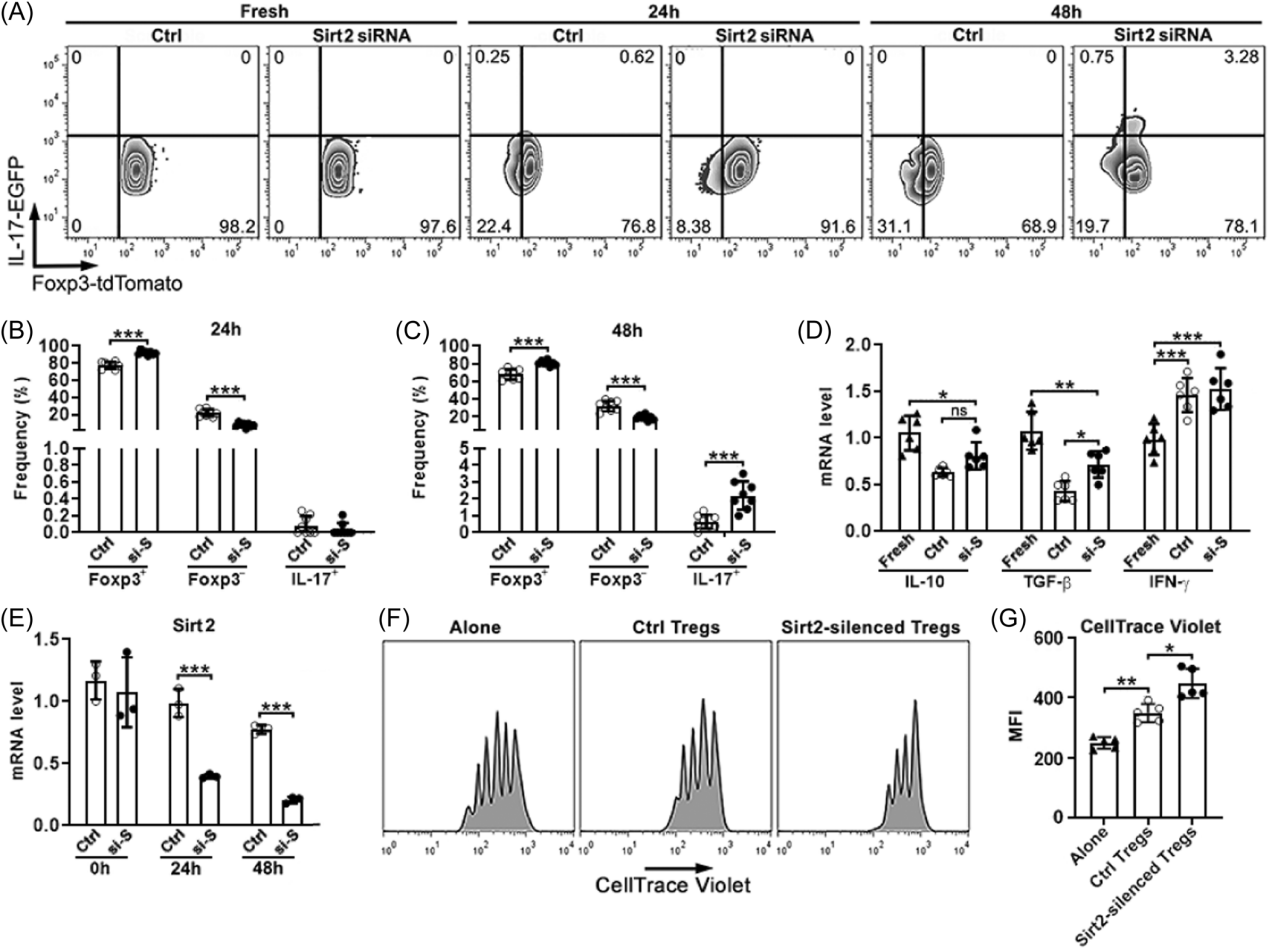
The effect of Sirt2 silencing on regulatory T cell (Treg)/T helper 17 (Th17) generation without stimulation. (A) Representative flow cytometry zebra plots showing the expression of interleukin (IL)‐17‐EGFP and Foxp3‐tdTomato in sorted lamina propria CD4^+^ tdTomato^+^EGFP^−^ Tregs after lentiviral transduction. 24 h, 24 h after transduction; 48 h, 48 h after transduction; Ctrl, control lentivirus encoding a scrambled siRNA; Fresh, freshly sorted Tregs; Sirt2 siRNA, Sirt2 siRNA‐encoding lentivirus. (B and C) The frequencies of Foxp3‐tdTomato^+^, Foxp3‐tdTomato^−^, and IL‐17‐EGFP^+^ cells in Tregs 24 h (B) or 48 h (C) after lentiviral transduction. Ctrl, cells transfected with control lentivirus; Si‐S, cells transfected with Sirt2 siRNA‐encoding lentivirus. (D) Messenger RNA (mRNA) levels of indicated cytokines 48 h after lentiviral transduction. Fresh: freshly sorted Tregs. (E) Sirt2 mRNA levels after transduction. (F) Representative histograms showing the dilution of CellTrace Violet in activated conventional CD4^+^ T cells after 5‐day coculture with Tregs. Alone: conventional CD4^+^ T cells alone. Ctrl Tregs: conventional CD4^+^ T cells co‐cultured with control Tregs. Sirt2‐silenced Tregs: conventional CD4^+^ T cells co‐cultured with Sirt2‐silenced Tregs. (G) Statistics of CellTrace Violet intensities. *N* = three to six per group. *p < .05; **p < .01; ***p < .001. Student's *t* test for (B), (C), and (E). One‐way analysis of variance for (D) and (G).

We sorted lamina propria CD4^+^ tdTomato^+^EGFP^−^ Tregs from healthy mice to test Sirt2 function because these Tregs highly express Sirt2. These Tregs were transduced with Sirt2 siRNA‐encoding lentivirus or control lentivirus encoding a scrambled siRNA, respectively. Twenty‐four to forty‐eight hours after transduction, the intensities of tdTomato and EGFP were evaluated. As shown in Figure 4A–C, compared with freshly sorted Tregs, tdTomato^+^ cells were decreased in both control Tregs and Sirt2‐silenced Tregs after incubation. However, Sirt2‐silenced Tregs had more tdTomato^+^ cells than control Tregs at each time point. Importantly, 48 h after transduction, Sirt2‐silenced Tregs generated more EGFP^+^ cells than control Tregs. Most of the EGFP^+^ cells were still tdTomato^+^, suggesting that they were Treg/Th17 cells co‐expressing Foxp3 and IL‐17. Furthermore, Sirt2‐silenced Tregs expressed higher TGF‐β than control Tregs when both groups downregulated the expression of IL‐10 and TGF‐β in comparison to freshly isolated Tregs (Figure 4D). Interferon‐gamma (IFN‐γ) expression was equivalently increased in both groups relative to freshly isolated Tregs (Figure 4D). Sirt2‐silenced Tregs and control Tregs equivalently upregulated IFN‐γ expression (Figure 4D). Sirt2 silencing was confirmed by q‐RT‐PCR (Figure 4E) and it did not influence Treg death (Supporting Information S1: Figure 3). Therefore, Sirt2 suppresses the expression of both Foxp3 and IL‐17 in Tregs. To verify the role of Sirt2, CellTrace Violet‐labeled conventional CD4^+^ T cells were co‐cultured with control Tregs or Sirt2‐silenced Tregs in the presence of agonistic antibodies for 5 days, followed by determining CellTrace Violet dilution in conventional CD4^+^ T cells. Both control Tregs and Sirt2‐silenced Tregs inhibited the proliferation of conventional CD4^+^ T cells, as evidenced by less CellTrace Violet dilution in conventional CD4^+^ T cells (Figure 4F,G). However, after co‐culture with Sirt2‐silenced Tregs, conventional CD4^+^ T cells exhibited a higher CellTrace Violet intensity compared with conventional CD4^+^ T cells co‐cultured with control Tregs, implying that Sirt2‐silenced Tregs were more immunosuppressive than control Tregs (Figure 4F,G).

### 3.5 The effect of Sirt2 silencing on Treg polarization to Th17 cells

**Figure 5:**
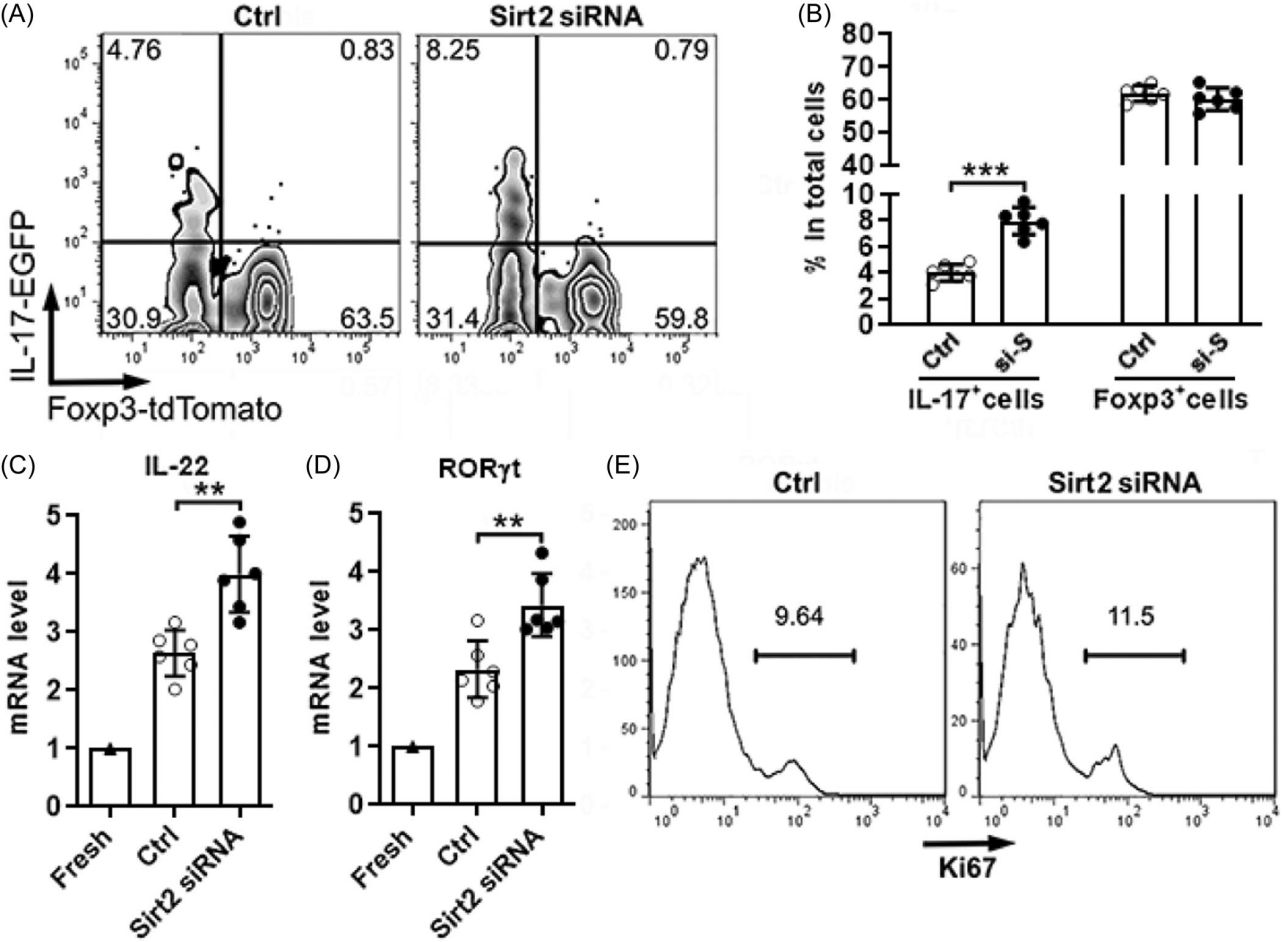
The effect of Sirtuin2 (Sirt2) silencing on regulatory T cells (Tregs) after T helper 17 (Th17) induction. (A) Representative flow cytometry zebra plots showing the expression of interleukin (IL)‐17‐enhanced green fluorescent protein (EGFP) and and Forkhead box P‐3 (Foxp3)‐tdTomato in sorted lamina propria CD4^+^ tdTomato^+^EGFP^−^ Tregs after lentiviral transduction and Th17 induction. Ctrl, cells transduced with control lentivirus; Si‐S, cells transduced with Sirt2 siRNA‐encoding lentivirus. (B) The frequencies of IL‐17‐EGFP^+^ cells and Foxp3‐tdTomato^+^ cells in Tregs after lentiviral transduction and Th17 induction. (C, D) Messenger RNA levels of IL‐22 (C) and retinoic‐acid‐receptor‐related orphan nuclear receptor gamma (D) in Tregs after lentiviral transduction and Th17 induction. Fresh: freshly sorted Tregs as the negative control. (E) Representative flow cytometry histograms showing Ki67 stain in Tregs after lentiviral transduction and Th17 induction. The data represent two independent experiments. *N* = 6 per group. **p < .01; ***p < .001. Student's *t* test.

To further check the role of Sirt2 in Treg plasticity, lamina propria CD4^+^ tdTomato^+^EGFP^−^ Tregs were first transduced with lentivirus. tdTomato^+^ cells were then resorted from the transduced cells by flow cytometry to exclude potential non‐Treg cells (Supporting Information S1: Figure 4). The sorted tdTomato^+^ cells were then subjected to Th17 induction. Three days after culture in the Th17 differentiation‐promoting condition, Sirt2‐silenced Tregs gave birth to more IL‐17‐EGFP^+^ Th17 cells than control Tregs (Figure 5A,B). However, the frequencies of the remaining Foxp3‐tdTomato^+^ cells were equivalent in Sirt2‐silenced Tregs and control Tregs (Figure 5A,B). Consistently, Sirt2‐silenced Tregs expressed more IL‐22 and RORγ than control Tregs after culture in the Th17 differentiation‐promoting condition (Figure 5C,D). Both groups exhibited comparable Ki67 staining, indicating that Sirt2 had no impact on Treg proliferation (Figure 5E).

### 3.6 The effect of Sirt2 silencing on the Stat3 signaling in lamina propria Tregs

**Figure 6:**
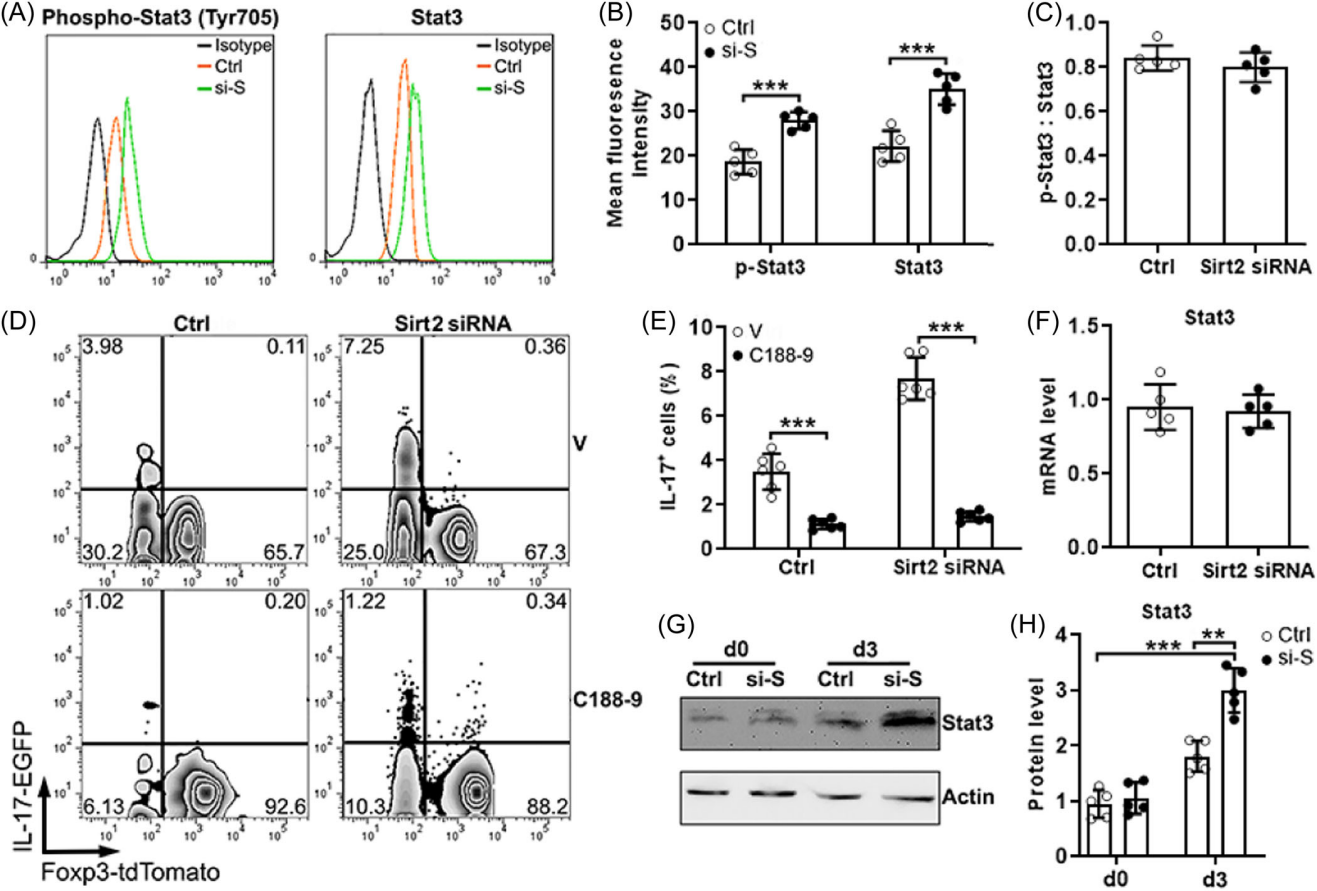
Involvement of the Sirtuin3 (Sirt3) signaling in Sirtuin2 (Sirt2)‐mediated effect. (A) Representative flow cytometry histograms showing the expression of phosphorylated Stat3 (left panel) and total Sta3 (right panel) in regulatory T cells (Tregs) after lentiviral transduction and T helper 17 (Th17) induction. Ctrl, cells transduced with control lentivirus; Isotype, isotype antibody control; Si‐S, cells transduced with Sirt2 siRNA‐encoding lentivirus. (B) Statistics of the mean fluorescent intensities of phosphorylated Stat3 (p‐Stat3) and total Stat3 in Tregs in (A). (C) The ratios of phosphorylated Stat3 (p‐Stat3) to total Stat3 in Tregs in (A). (D) Representative flow cytometry zebra plots showing the expression of interleukin (IL)‐17‐EGFP and Foxp3‐tdTomato in Tregs after lentiviral transduction and Th17 induction in the presence or absence of C188‐9. V: DMSO. (E) Statistics of the frequencies of IL‐17‐EGFP^+^ cells in (D). (F) Sirt2 messenger RNA levels in Tregs after lentiviral transduction and Th17 induction. (G) Immunoblotting images showing Stat3 proteins in Tregs on Day 0 (d0) and Day 3 (d3) after Th17 induction. (H) Statistics of normalized Stat3 protein levels in (G). *N* = five to six per group. ***: *p* < .001. Student's *t* test. DMSO, dimethyl sulfoxide.

Because the Stat3 signaling is crucial for Th17 induction, we conducted the phos‐flow assay to analyze the levels of both phosphorylated Stat3 (Tyr705) and total Stat3 in lamina propria tdTomato^+^EGFP^−^Tregs after lentiviral transduction and Th17 induction. As shown in Figure 6A,B, Sirt2‐silenced Tregs expressed higher phosphorylated Stat3 as well as total Stat3 than control Tregs. However, the ratios of phosphorylated Stat3 to total Stat3 were comparable in both groups, indicating that Stat3 phosphorylation itself was not affected by Sirt2 silencing (Figure 6C). To confirm the role of Stat3 in the Sirt2‐mediated effect, C188‐9, a selective and potent Stat3 inhibitor, was used to treat Tregs under the Th17 induction condition. C188‐9 profoundly abolished the generation of IL‐17^−^EGFP^+^ Th17 cells from not only control Tregs but also Sirt2‐silenced Tregs (Figure 6D,E). To unveil the mechanism by which Sirt2 inhibits Stat3 expression, we first analyzed Stat3 mRNA levels but found no impact of Sirt2 silencing on Stat3 mRNA quantity (Figure 6F). The Immunoblotting assay confirmed more Sta3 protein in Sirt2‐silenced Tregs relative to control Tregs after Th17 induction, suggesting that Sirt2 might suppress Stat3 translation, degradation, or recycling in Tregs (Figure 6G,H).

## 4 DISCUSSION

The current research provides valuable insights into the significance of Sirt2 to Tregs in inflammatory colitis. The new Foxp3‐tdTomato‐IL17^−^EGFP mouse strain is a good tool for simultaneously monitoring Tregs, Th17 cells, and Treg/Th17 cells in not only colitis but other disorders. Particularly, we identified high expression of Sirt2 in Tregs of normal lamina propria. Interestingly, in comparison to Tregs of normal lamina propria, Tregs of colitic lamina propria downregulated Sirt2. Considering the importance of Tregs for curbing inflammatory response, it is plausible to deduce that Sirt2 could play a notable role in lamina propria Treg function. However, the mechanisms underlying the high Sirt2 expression in lamina propria Tregs remain unclear. A previous study indicates that poststroke microglia induce Sirt2 expression in infiltrating Treg cells through hypoxia‐inducible factor‐1α.^17^ It is, therefore, possible that macrophages or dendritic cells induce Sirt2 expression in Tregs in normal lamina propria whereas colitis‐related inflammatory mediators inhibit Sirt2 expression. Based on this hypothesis, our next project will dissect the factors responsible for Sirt2 upregulation and downregulation. Besides, other Sirtuins, such as Sirt4 and Sirt6, were also found to be highly expressed in either normal or colitic lamina propria. Therefore, although we focused on Sirt2, Sirt4, and Sirt6 should be investigated in the same model in the future.

This study also showed that Sirt2 was predominantly expressed in Foxp3^+^IL‐17^−^Tregs whereas Foxp3^+^IL‐17^+^ Treg/Th17 cells in colitic lamina propria. Interestingly, in colitic mesenteric lymph nodes, Foxp3^+^IL‐17^+^ Treg/Th17 cells expressed less Sirt2 than Foxp3^+^IL‐17^−^Tregs even though the latter did not express high Sirt2. This phenomenon suggests that Sirt2 might be important for Th17 differentiation or function. Indeed, we found that Sirt2 silencing increased the expression of IL‐17, IL‐22, and RORγ expression in Tregs with or without Th17 induction, confirming the inhibitory effect of Sirt2 on Treg/Th17 differentiation. Moreover, we identified Stat3 as a key factor involved in the Sirt2‐mediated effect, as evidenced by downregulating both phosphorylated Stat3 and total Stat3 in Sirt2‐silenced Tregs under Th17 induction. Th17 cell development has been reported to rely on Stat3 signaling activation under the instruction of key cytokines including IL‐6, IL‐21, and IL‐23.^20^, ^21^, ^22^ Interestingly, Stat3 also programs Th17 lineage‐specific Tregs (another name for Treg/Th17 cells) in crescentic glomerulonephritis which is an inflammatory disorder.^23^ Therefore, it is very likely that Stat3 also favors the formation of Treg/Th17 cells and Sirt2 decreases Stat3 to mitigate Treg/Th17 cell differentiation. Besides, our group is still studying whether Sirt2 suppresses Th17 development and function in lamina propria because Th17 differentiation and function also depend on Stat3.

Another finding of this study is that Sirt2 undermines Treg function, as evidenced by higher expression of Foxp3 and TGF‐β in Sirt2‐silenced Tregs without Th17 induction. This result is consistent with the previous study displaying the inhibitory effect of Sirt2 on Treg cells in a stroke model.^17^ It is thus reasonable to conclude that Sirt2 is a negative regulator of Tregs. Additionally, it would be interesting to explore the expression patterns and functions of Sirt2 in other T helper populations such as Th1, Th2, and Th9 cells in future studies.

Sirt2 is an NAD^+^‐dependent deacetylase that deacetylates internal lysines of proteins including several transcription factors.^24^ However, how Sirt2 impacts the expression of Foxp3 and Stat3 is unknown. Sirt2 is reported to inhibit the nuclear factor kappa B (NF‐κB) activity^24^ and NF‐κB is crucial for Foxp3 expression during Treg cell development.^25^ Sirt2 supports optimal Akt activation^26^ and Akt suppresses Foxp3 expression.^27^ Sirt2 perhaps functioned via the same mechanisms in our experimental settings. However, little is known about the Sirt2‐mediated mechanism for the change of Stat3. Our study suggests that Sirt2 reduces Stat3 protein rather than Stat3 mRNA. Therefore, future studies should pay attention to Stat3 translation, posttranslational modification, degradation, and recycling.

Notably, Sirt2 seems to inhibit both Treg function and Treg/Th17 cell generation, implying that Sirt2 could be a double‐edged sword in the context of colitis. On one hand, Tregs and Treg/Th17 cells can be immunosuppressive to maintain immune homeostasis.^28^, ^29^ Sirt2 upregulation might impair the anti‐inflammatory and immunosuppressive activity of Tregs to exacerbate autoimmune colitis. On the other hand, Treg/Th17 cells, which produce pro‐inflammatory IL‐17, might contribute to colitis pathogenesis as they do in colon cancer and allergic asthma.^30^, ^31^ In this regard, Sirt2 would prevent Treg/Th17 cell generation to curb colitis. The exact role of Sirt2 in colitis development presumably depends on the extent of autoimmunity and inflammation. In the early stage of colitis when bowel inflammation is mild, Sirt2 expression could undermine Treg function and thus should be inhibited. When colitis becomes severe and cumulative pro‐inflammatory cytokines drive Tregs to differentiate into Treg/Th17 cells, Sirt2 expression would alleviate pathogenic Treg/Th17 cell generation to thus favor the resolution of colitis. If this is the case, therapeutic manipulation of Sirt2 expression, either promotion or inhibition, should be conducted in a temporal pattern. Future investigations using inducible Treg‐specific and Treg/Th17‐specific Sirt2 knockout or knock‐in models will provide insights into the significance of Sirt2 in colitis.

This study still has several limitations: (1) The findings are based on a mouse model and need to be verified by human samples. (2) The mechanisms underlying the high Sirt2 expression in lamina propria Tregs and the associated change in colitis remain unclear. (3) The temporal change of Sirt2 in Tregs during different colitis stages is still not clear. Samples of mild, moderate, severe, and remission stages should be analyzed in the future. (4) The role of Sirt2 in Treg function and Treg/Th17 differentiation was only analyzed in vitro and lacks in vivo data. (5) The impact of Sirt2 expression on colitis development remains obscure. And whether Sirt2 silencing influences colitis progression is unclear. (6) Whether Sirt2 exerts similar effects in other autoimmune disorders needs to be assessed in the future.

**Figure 7:**
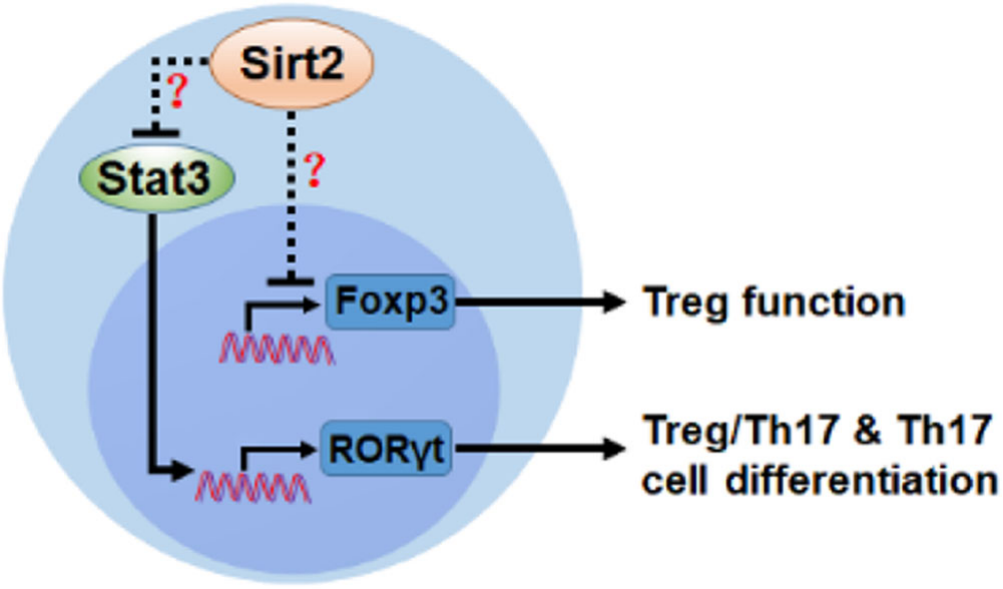
Overview of the findings. Foxp3, Forkhead box P‐3; Sirt2, Sirtuin2; Sirt3, Sirtuin3; RORγt, retinoic‐acid‐receptor‐related orphan nuclear receptor gamma T; Th17, T helper 17; Treg, regulatory T cell.

In summary, Sirt2 suppresses Treg function through unidentified mechanisms and inhibits Treg polarization toward Th17 cells via downregulating Stat3 expression (Figure 7). Sirt2, therefore, could either exacerbate or improve colitis progression, depending on the delicate balance among Tregs and Treg/Th17 cells (and even Th17 cells). Our team will construct a Treg‐ or Th17‐specific Sirt2 knockout model to identify the precise roles of Sirt2 in the modulation of functions of Tregs and Th17 cells.

## AUTHOR CONTRIBUTIONS

**Liuqing Ge**: Conceptualization; investigation; methodology. **Min Xu**: Data curation; Investigation; methodology. **Meifang Huang**: Investigation. **Shaoping Liu**: Investigation. **Zhidai Zhou**: Investigation. **Ziqin Xia**: Investigation. **Qiu Zhao**: Methodology. **Feng Zhou**: Supervision, validation; writing—original draft preparation; writing—review and editing funding acquisition.

## CONFLICT OF INTEREST STATEMENT

The authors declare no conflict of interest.

## ETHICS STATEMENT

The study was approved by the Wuhan University Animal Care and Use Committee and implemented under the Wuhan University Animal Use Guidelines (Approval# 11400700245566).

## Supporting information

Supporting information.

## Data Availability

The data are available from the corresponding author upon reasonable request.
