# Supplementary material for: Sirtuin2 suppresses the polarization of regulatory T cells toward T helper 17 cells through repressing the expression of signal transducer and activator of transcription 3 in a mouse colitis model
Source: Immun Inflamm Dis. 2024 Feb 2;12(2):e1160. doi: 10.1002/iid3.1160 (PMC10836035; doi:10.1002/iid3.1160)
Supplement: Supplementary file 1 — Supporting information. [file IID3-12-e1160-s001.docx]

**Supplementary information**

|  | | | |
| --- | --- | --- | --- |
| **Supplementary Table 1. Primer Sequences** | | | |
| **Target** | **Sense (5’ to 3’)** | | **Antisense (5’ to 3’)** |
| Sirt1 | TACCTTGGAGCAGGTTGCAG | GCTTCATGATGGCAAGTGGC | |
| Sirt2 | TTTGGTGGGAGCCGGAATC | CCAGGTTTGCATAGAGGCCA | |
| Sirt3 | TCCGGGAGGTGGGAGAAG | CACCATGACCACCACCCTAC | |
| Sirt4 | TGAAAGAGGCGGACTCCCTA | CAGGCAAGCCAAATCGTCA | |
| Sirt6 | GCCCAACAGCCCTATACTCC | TGTGGTTCCTTCAAGTTCCCC | |
| IL-10 | AGGCGCTGTCATCGATTTCT | ATGGCCTTGTAGACACCTTGG | |
| IFN-γ | CAGCAACAGCAAGGCGAAAAAGG | TTTCCGCTTCCTGAGGCTGGAT | |
| TGF-β | CTGCTGACCCCCACTGATAC | GTGAGCGCTGAATCGAAAGC | |
| IL-22 | GCTTGAGGTGTCCAACTTCCAG | ACTCCTCGGAACAGTTTCTCCC | |
| RORγ | GTGGAGTTTGCCAAGCGGCTTT | CCTGCACATTCTGACTAGGACG | |
| Stat3 | AGGAGTCTAACAACGGCAGCCT | GTGGTACACCTCAGTCTCGAAG | |
| Actin | GATGGTGAAGGTCGGTGTGA | TGAACTTGCCGTGGGTAGAG | |

**
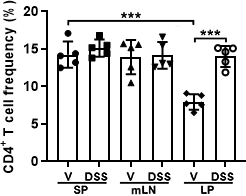
**

**Supplementary Figure 1. The frequencies of CD4^+^ T cells in the spleens (SP), mesenteric lymph nodes (mLN), and lamina propria (LP).** V: vehicle-treated mice. DSS: DSS-treated colitic mice. N=6 per group. ***: P < 0.001. One-way ANOVA.

**
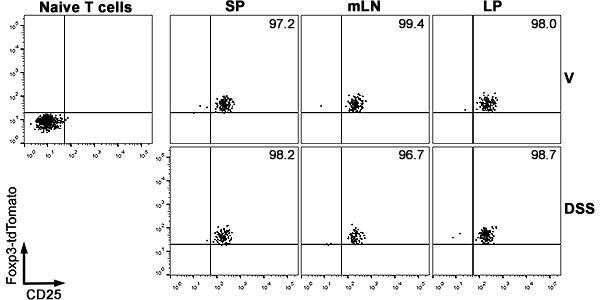
**

**Supplementary Figure 2. The purity of sorted Tregs.** CD4^+^tdTomato^+^ Tregs were sorted from the spleen (SP), mesenteric lymph nodes (mLN), and lamina propria (LP) by a sorter and then re-analyzed on a flow cytometer. Some CD3^+^CD4^+^CD25^-^tdTomato^-^ naive T cells were also sorted as the negative control. V: vehicle-treated mice. DSS: DSS-treated colitic mice.

**
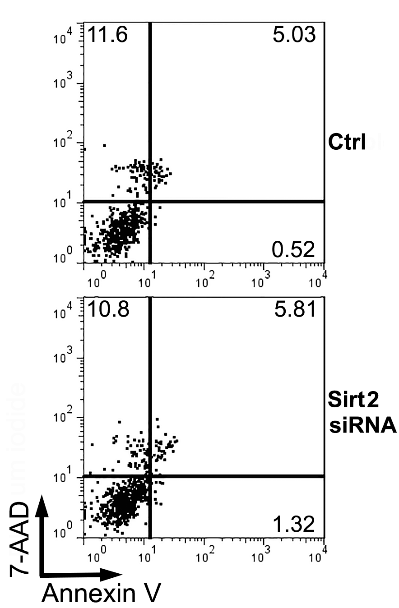
**

**Supplementary Figure 3. Apoptotic (Annexin V^+^) and necrotic (7-AAD^+^) Tregs 48 hours after lentiviral transduction.** The data represent 2 independent experiments. Control: control lentivirus encoding a scrambled siRNA. Sirt2 siRNA: Sirt2 siRNA-encoding lentivirus.

**
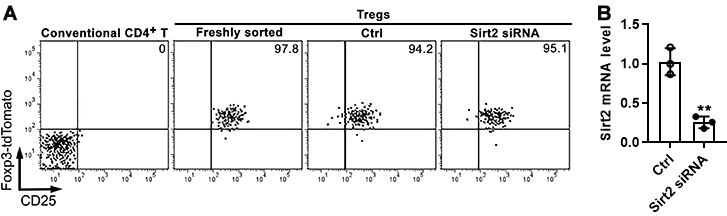
**

**Supplementary Figure 4. (A) The purity of Foxp3^+^ Tregs after tdTomato^+^ cells were sorted from lentivirus-transduced Tregs.** CD4^+^tdTomato^+^EGFP^-^ Tregs were first transduced with the control lentivirus (Ctrl) or Sirt2 siRNA-encoding lentivirus (Sirt2 siRNA) overnight and incubated in fresh medium for 2 additional days. tdTomato^+^ cells, which represented cells that maintained Treg identity, were sorted by flow cytometry from the transduced cells to exclude non-Treg cells, followed by testing the purity of Foxp3^+^ Tregs. Conventional CD4^+^CD25^-^ T cells (Conventional CD4^+^ T) were used as the negative control while freshly sorted non-transduced lamina propria CD4^+^tdTomato^+^ Tregs (freshly sorted) were used as the positive control. **(B)** Sirt2 mRNA levels in tdTomato^+^ Tregs that were sorted from lentivirus-transduced Tregs. N=3 per group. **: P < 0.01.
